# Development and Validation of an Algorithm for Item Reduction of the International Standards for Neurological Classification of Spinal Cord Injury Examination to Determine Level and Severity of SCI

**DOI:** 10.46292/sci25-00008

**Published:** 2025-08-22

**Authors:** Stephen P. Burns, Kristen Walden, Steven Kirshblum, Mary Schmidt-Read, Keith Tansey, Christian Schuld, Ruediger Rupp

**Affiliations:** 1 Spinal Cord Injury Service, VA Puget Sound Health Care System, Seattle, Washington; 2 Department of Rehabilitation Medicine, University of Washington School of Medicine, Seattle, Washington; 3 Praxis Spinal Cord Institute, Vancouver, British Columbia, Canada; 4 Kessler Institute for Rehabilitation, West Orange, New Jersey; 5 Rutgers New Jersey Medical School, Newark, New Jersey; 6 Jefferson-Moss-Magee Rehabilitation, Philadelphia, Pennsylvania; 7 Spinal Cord Injury Clinic, Jackson VA Medical Center, Jackson, Mississippi; 8 Departments of Neurosurgery, University of Mississippi Medical Center, Jackson, Mississippi; 9 Spinal Cord Injury Center, Heidelberg University Hospital, Heidelberg, Germany; 10 Medical Faculty Heidelberg, Heidelberg University, Heidelberg, Germany

**Keywords:** classification, humans, physical examination, spinal cord injuries

## Abstract

**Background:**

In 2020, a first, expedited version of the International Standards for Neurological Classification of Spinal Cord Injury (E-ISNCSCI-V1) was proposed for determination of neurological level of injury (NLI) and American Spinal Injury Association Impairment Scale (AIS) classifications.

**Objectives:**

This work describes assessment of E-ISNCSCI-V1 classification accuracy and the development and data-based validation of an ISNCSCI Item Reduction Algorithm (IIRA).

**Methods:**

Classification accuracy for E-ISNCSCI-V1 examination shortcut options was assessed with automated analysis of 7026 full ISNCSCI examinations. Rules for the IIRA were iteratively adjusted to optimize the balance between omitting exam items and minimizing misclassification errors, and then it was validated through classification of 100 full ISNCSCI exams.

**Results:**

If S1 findings are substituted for anorectal exam findings as proposed for E-ISNCSCI-V1, the error rate for AIS is 10%, with a high error rate (45%) for classifying true AIS B. The IIRA, which begins with full motor testing, followed by limited sensory testing required an average of 31% (42/134) of the full ISNCSCI exam items, with a 2% error rate for NLI and no AIS errors.

**Conclusion:**

The previously proposed E-ISNCSCI-V1, which included an option to substitute S1 findings for anorectal exam findings, is not recommended due to AIS error rate. The IIRA provides a standardized option for a shortened examination classifying NLI and AIS with high accuracy. It will serve as a basis for version 2 of the E-ISNCSCI.

Spinal cord injury (SCI) produces patterns of neurological impairments due to the segmental structure of the spinal cord and the variable severity of sensorimotor deficits caused by injury or disease. For more than 35 years, the International Standards for Neurological Classification of Spinal Cord Injury (ISNCSCI) has been widely accepted as the preferred method for classification of neurological impairment.^1^ The 2 most widely used ISNCSCI classification variables for basic characterization are the neurological level of injury (NLI) and the American Spinal Injury Association (ASIA) Impairment Scale (AIS), which reflect the level and severity of the spinal lesion, respectively.

ISNCSCI completion (the “full ISNCSCI”) requires testing of every defined exam component: light touch (LT) and pinprick (PP) sensation (sharp-dull discrimination) testing of 56 dermatomes (28 on each side); manual muscle testing of 20 upper and lower extremity key muscles (10 on each side) plus additional non-key muscles in some cases; and digital anorectal exam for deep anal pressure (DAP) and voluntary anal contraction (VAC).^1^ It thus requires testing and documentation of at least 134 exam items. The time required, especially for the sensory exam, represents a high burden for patients and examiners. Clinicians therefore often perform nonstandardized examinations or no examinations at all. Finally, for multiple reasons, the requirement for an anorectal exam is another barrier and is one of the most commonly omitted exam items.^2^

The ASIA International Standards Committee recognized the need for an option to rapidly determine NLI and AIS when the full ISNCSCI cannot feasibly be performed or if the accuracy of the full ISNCSCI is not necessary. A protocol for version 1 of an expedited ISNCSCI exam, or E-ISNCSCI-V1, was developed and posted to the ASIA website in 2020 along with 4 videos demonstrating classifications.^3^,^4^ The E-ISNCSCI-V1 protocol contains general instructions on a testing sequence to identify the NLI and AIS with a minimum number of exam items. It also includes options to substitute S1 sensory and motor findings in place of the anorectal exam and to omit upper extremity motor testing for some cases, based upon prior recommendations.^5^ No systematic evaluation of E-ISNCSCI-V1 classification accuracy has been published. Additionally, the ASIA International Standards Committee received feedback that the protocol wording was unclear and that clinically important information (i.e., the motor scores of all 20 key muscles) should be included.

This work describes the data-based evaluation of the E-ISNCSCI-V1, including the accuracy of AIS classification when S1 is substituted for anorectal exam findings. Based on those results, a new algorithm for reduction of the number of ISNCSCI exam items required for NLI and AIS determination was iteratively developed, and its data-based validation is presented.

## Methods

In E-ISNCSCI-V1, the protocol for performing sensory testing was to complete testing “sufficiently to identify the most caudal intact dermatomes.”^3^ The imprecise instructions thus prevented determination of NLI classification accuracy or total exam items required. E-ISNCSCI-V1 also described 2 “shortcut options”: omitting upper extremity testing if the most rostral sensory level was T4 or caudal, and substituting S1 findings for anorectal exam findings for AIS determination if the NLI was T10 or rostral. Classification accuracy for the 2 shortcut options was modeled using retrospective analysis of deidentified full ISNCSCI exams performed on Rick Hansen Spinal Cord Injury Registry (RHSCIR) participants between 2004 and 2022. The RHSCIR is a Canadian national prospective registry of adults with new SCI admitted to one of 30 acute care and rehabilitation facilities across Canada.^6^,^7^ The dataset used for this study included 7026 full ISNCSCI case examinations (“cases”) performed on 4050 individuals with traumatic SCI (mean 1.73 exams per individual), with median injury duration of 10.5 days at examination. Mean age was 48.5 years (*SD* 19.2 years; age range 15-96 years), 78.4% were male, and the most common injury etiologies were falls (44.6%) and transportation (28.7%). Clinicians had initially classified the cases, which were subsequently checked for classification accuracy using a computerized algorithm^8^,^9^ and corrected if necessary. For the 7026 cases, NLI was cervical (60.9%), thoracic (27.4%), and lumbosacral (11.7%), and AIS grade was A (33.5%), B (11.5%), C (15.9%), and D (39.2%).

The analyses for NLI classification accuracy when omitting upper extremity motor testing determined the frequency of any motor deficit in C5-T1 myotomes for 3665 cases with most rostral sensory level of T2 and caudal. If upper extremity weakness was present in these cases and motor testing was omitted, the NLI classification would be incorrect. For the analysis of S1 substitution, in alignment with previously published work,^9^ an S1 sensory score of 1/2 or 2/2 in either LT or PP on the right or left side was substituted for a score of “yes” for DAP and presence of sensation at S4-5, whereas an S1 sensory score of 0/2 in both LT and PP on both right and left side resulted in DAP score of “no” and absence of sensation at S4-5. An S1 motor score of 1/5 through 5/5 on left or right side was substituted for VAC presence score of “yes,” with a score of 0/5 on both sides substituted as “no” for VAC. We calculated rates for all misclassifications between three categories of AIS grades: A, B, and merged C and D. For example, we identified all cases with true AIS C or D that would be incorrectly classified as AIS A as follows: for all cases with true AIS C or D, the proportion of those with bilaterally S1 LT and PP = 0/2 and S1 motor = 0/5 were identified. See eTable 1 for criteria used to calculate misclassification rates. We also determined the rate of incorrect injury completeness (AIS A vs. B, C or D) at each NLI from C1 through S3.

Final analyses, used to develop a new classification algorithm (ISNCSCI Item Reduction Algorithm [IIRA]) based on expert panel recommendations, included the minimum number of exam items necessary to determine NLI and AIS and the accuracy of NLI and AIS determinations. Analyses used a random sample of deidentified full ISNCSCI case examinations derived from the European Multicenter Study about SCI (EMSCI) database.^10^ Cases were excluded if any exam items were recorded as “not testable” or if non-SCI conditions were documented. An algorithm development sample of 20 randomly selected full ISNCSCI cases was created, and each case was classified by at least 2 of the 6 expert investigators who counted the minimum number of exam items needed to establish NLI and AIS. Each expert investigator was a current member of the ASIA International Standards Committee, had previously completed formal ISNCSCI training courses sponsored by ASIA, and had participated as an instructor in ASIA's ISNCSCI training courses. The IIRA was then revised to eliminate ambiguous wording and improve the accuracy of the determined classifications based on the specific reasons that classification errors occurred in the first sample of cases. Next, 5 of the expert investigators each classified 20 additional cases (100 cases total) using the updated draft IIRA. The minimum number of exam items needed was counted for each case. Accuracy of NLI determinations was assessed by comparing them with the NLI derived from the full ISNCSCI exam. Based on a high rate and magnitude of NLI errors using the initial algorithm, further algorithm modification options were assessed on cases with NLI errors. The sequences of exam testing (LT, PP, and motor) and the amount of motor testing performed (omission of upper limb testing, partial motor testing until first abnormal myotomes are identified, full motor testing) were altered to optimize NLI accuracy. Inclusion of full motor testing was chosen because the expert examiners recognized this would have clinical utility and allow calculation of summed motor scores for all cases, in addition to simplifying the teaching of the testing sequence. The final modification of the IIRA that minimized NLI error was then tested for accuracy on a validation sample of 100 additional full ISNCSCI cases from EMSCI, each of which was classified twice by the investigators.

## Results

### NLI classification accuracy

Analysis of the 7026 cases from the RHSCIR database confirmed that upper extremity weakness was more common with sensory levels of T2 (44%) and T3 (20%), but upper extremity weakness was present for 10% or more of cases in some sensory level groupings T4 or caudal (see eFigure 1). Such cases therefore would receive inaccurate NLI classifications if upper extremity motor testing were skipped.

NLI classification accuracy using the preliminary version of IIRA was assessed on 100 full ISNCSCI cases from the EMSCI database. Ten cases were classified incorrectly for NLI, with a mean discrepancy of 10.4 (*SD* 8.1) levels between the correct and incorrect NLI. Cases with NLI errors were reclassified using alternate examination sequences, and the sequence with highest accuracy was selected.

The final version of this newly developed algorithm (IIRA) is included in the eAppendix, along with 2 case classification examples. Using the IIRA, 9 of 10 cases that produced NLI errors using the preliminary algorithm were correctly classified, with an error of 3 levels (C8 vs. C5) for NLI determination in the one case with a discrepancy. Next, the second sample of 100 full ISNCSCI cases was classified. Two of 100 cases showed NLI discrepancies, both with discrepancies of 6 levels (T12 vs. T6, and S1 vs. T12) between identified and correct NLI. Both cases had large side-to-side sensory level asymmetry, with multiple normal dermatomes located caudal to the isolated sensory deficit that determined the correct NLI.

### AIS classification accuracy

The initial analyses determined the classification error rates for the S1 substitution option described in E-ISNCSCI-V1. **Table 1** shows the distribution of correct and incorrect AIS classifications in the 7026 analyzed cases. Overall, 89.5% of cases were assigned the correct AIS grouping. When error rates are expressed based on true AIS, error rates vary considerably between the different AIS groupings. True AIS A was correctly classified for 92.5% (2171/2347) of cases and true C/D was correctly classified for 94.9% (3677/4024) of cases. In contrast, true AIS B was classified correctly for only 55% (442/803) of cases, with misclassifications most commonly resulting in AIS A.

**Table 1.** AIS determinations with use of S1 substitution shortcut

True AIS	AIS with S1 substitution		
	A	B	C/D
**A**	2171 (30.9%)	51 (0.7%)	125 (1.8%)
**B**	338 (4.8%)	442 (6.3%)	23 (0.3%)
**C/D**	161 (2.3%)	38 (0.5%)	3677 (52.3%)

*Note:* AIS = American Spinal Injury Association Impairment Scale.

When error rates are expressed based on the AIS determined by use of the S1 substitution, there is also substantial variation in error rates seen across AIS groupings. For cases classified as AIS A using S1 substitution, only 81.3% (2171/2670) are true AIS A; for cases determined to be AIS B using the substitution, only 83.2% (442/531) are true AIS B. When use of the S1 substitution indicates that the case is AIS is C or D, this was correct for 96.1% (3677/3825) of cases.

**Figure 1.**
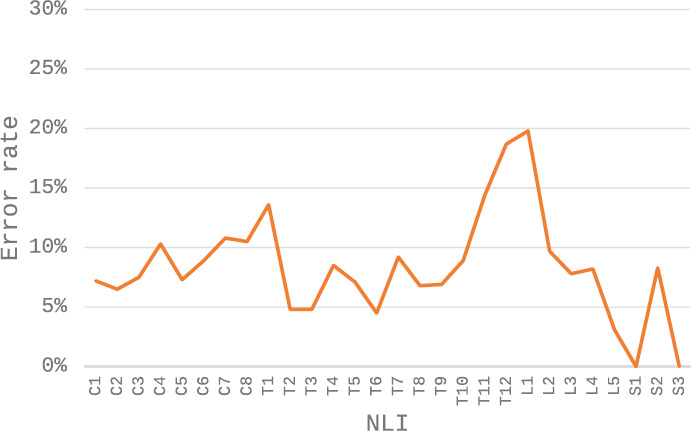
The error rate is the percentage of cases at each neurological level of injury (NLI) that would be assigned an incorrect injury completeness classification using S1 substitution.

**Figure 1** shows the error rate for determination of injury completeness (complete = AIS A, incomplete = AIS B, C or D) at each true NLI. The highest error rates (18.7% and 19.8%) were for NLI of T12 and L1. Error rates were above 10% at C4, C7, C8, and T1 and were under 10% at all NLI caudal to L1. Based on this distribution of classification errors versus NLI, the recommendation of E-ISNCSCI-V1 to consider using S1 substitution if the NLI was T10 or rostral was not supported; substantial error rates occur at common cervical levels, with lower error rates at NLI of L1 and caudal.

Finally, accuracy of AIS classification using the final version of the IIRA, which allows omission of a portion of S4-5 testing and anorectal exam for some exams, was assessed in the validation sample of 100 EMSCI cases. The IIRA correctly identified AIS for all cases, including the 2 for which NLI was determined incorrectly.

### Reduction of the number of exam items

The final version of the IIRA, assessed on the validation sample of 100 EMSCI cases, required a mean of 41.9 (*SD* 9.2) exam items (31% of the 134 items of the full ISNCSCI) to classify NLI and AIS with high accuracy. For a typical case, the required 42 exam items would include 20 motor exam items, 21 sensory exam items, and one item of anorectal exam (DAP or VAC). On average, cases with cervical NLIs required 15.6 fewer exam items than cases with more caudal NLIs (mean number of 33.8 vs. 49.4 items). Cases with greater injury severity tended to require slightly more exam items: 45.7, 45.4, 40.4, and 37.2 items for AIS A, B, C, and D, respectively. The digital anorectal exam was not required for 44 of 100 cases of the validation sample.

## Discussion

Version 1 of the E-ISNCSCI protocol^3^ lacked specific testing sequence instructions and thus could not be assessed for either NLI accuracy or total exam items required. It included an option to use S1 sensory and motor for AIS determination, for which accuracy had not been evaluated. This study determined the accuracy of S1 substitution, developed and iteratively adjusted a new, detailed ISNCSCI item reduction algorithm (IIRA) optimizing the balance between omitting exam items and minimizing misclassification errors, and finally assessed its accuracy. The accuracy evaluations used automated analyses of a large examination database plus individual case classifications performed by expert investigators. The overall error rate for the S1 substitution option was 10.5%, but it had a 45% error rate for true AIS B cases, and 21% of all cases identified as AIS A were misclassifications. Thus, S1 substitution is not recommended as an option if an accurate AIS is needed, and it has therefore not been used for the IIRA. The new algorithm identified the correct NLI for 98 of 100 cases and the correct AIS for all 100 cases in the validation sample, while requiring only 31% of the exam items of the full ISNCSCI. Most skipped items consisted of sensory exam items.

Previous work on developing shortened examinations for SCI neurological classification has used a variety of approaches, either consistent with or dissimilar to the exam used for the full ISNCSCI.^3^,^9^,^11^-^13^ Development of computer algorithms for automatic classification of full ISNCSCI examinations^7^,^8^,^14^ reinforced what was recognized by clinicians and researchers: for NLI and AIS determinations, much of the sensory exam is not needed. Methods previously proposed to reduce sensory testing include standardized reduction of tested dermatomes and sensory modalities. These approaches were rejected for use in the IIRA because they would fail to precisely identify sensory findings that determine an NLI. The IIRA precisely localizes the NLI because the algorithm provides a consistent approach to which dermatomes may be omitted. A protocol was recently proposed that identifies a neurological level while eliminating all PP sensation testing and also omitting LT sensation testing at segments with testable motor functions (C5-T1 and L2-S1) plus S2 and S3.^13^ With that protocol, total exam components are reduced to 40% of the full ISNCSCI. The identified level was within 2 levels of the true NLI per ISNCSCI, but it exactly corresponded with the true NLI for only 71% of 35 cases. The IIRA provides presumptive identification of the NLI and AIS with much higher accuracy than the approaches described above.

The anorectal exam is one of the most commonly omitted portions of the full ISNCSCI.^2^ Prior research had established correlation between specific exam findings and the sacral sparing exam components (S4-5 sensation, DAP, and VAC) used for injury completeness and AIS determination.^9^,^11^ Thus, we assessed the accuracy of substituting S1 findings for determination of AIS, which was an option in E-ISNCSCI-V1 if the NLI was T10 or rostral.^3^ Our analysis showed that the highest error rates for complete versus incomplete determination were at T12 and L1, but error rates were lower at more caudal NLIs. Also, substantial error rates (10.3% and 13.6%) occurred at C4 and T1 NLI, respectively. Overall, the S1 shortcut resulted in incorrect AIS grouping for 10.5% of cases. Importantly, the highest misclassification rate was detected in AIS B cases, with only 55% classified correctly due to a high rate of incorrect classification as AIS A. Newly injured patients with AIS B have a markedly better prognosis for neurological and functional recovery than those with AIS A.^15^ For this reason, the S1 substitution is considered to have inadequate accuracy and is not recommended.

## Study Limitations

Although some study analyses were performed on a large data set of full ISNCSCI examinations, the final ones for IIRA accuracy were assessed using 2 different samples of 100 examinations each from traumatic or ischemic patients within their first year after SCI. Findings could differ for examinations performed on individuals with longer SCI duration or other nontraumatic etiologies. The frequency of upper extremity motor weakness for cases with sensory levels of T4 or caudal in both databases (RHSCIR and EMSCI) was higher than had been expected and caused NLI errors with E-ISNCSCI-V1. A possible reason is that examiners may have detected weakness of upper extremity muscles that was due to non-SCI conditions. However, a revised method for indicating presence of weakness due to non-SCI conditions in the ISNCSCI was only introduced with the 2019 revision of the ISNCSCI. 16 Although all classifications in the RHSCIR and EMSCI databases had previously been verified as correct using computerized algorithms, this process would not have detected incorrect exams or failure to recognize the presence of non-SCI conditions affecting exam findings. In a small percentage of cases for which the “motor follows sensory level” rule for NLI is required,^1^ the IIRA will not identify the motor level unless additional sensory testing is performed. This did not occur in any of the 100 validation cases.

It remains to be determined how difficult it will be for clinicians and researchers to learn the testing sequence and classification of the partial exam required for IIRA. As has been done with prior versions of the full ISNCSCI, accuracy of classifications based on this algorithm needs to be assessed in prospective studies. Such results, along with ongoing feedback from training workshops and suggestions from the field, will promote development of clear examiner instructions for a future version of the E-ISNCSCI and account for difficult classification cases not anticipated or encountered in the cases used for IIRA development.

## Conclusion

The E-ISNCSCI-V1, which included options to substitute S1 findings for anorectal exam findings and omit upper limb testing, is not recommended due to substantial errors in classifying AIS grades and NLI caused by those options, respectively. The newly developed ISNCSCI IIRA has high accuracy in determining the NLI and AIS, while on average requiring only 31% of the 134 exam items of the full ISNCSCI and allowing omission of the anorectal exam for 44% of cases. The IIRA will serve as a basis for development of version 2 of the E-ISNCSCI exam protocol.

## Supplementary Material







## References

[1] Rupp R, Biering-Sørensen F, Burns SP (2021). International Standards for Neurological Classification of Spinal Cord Injury: Revised 2019. Top Spinal Cord Inj Rehabil.

[2] Bourguignon L, Tong B, Geisler F (2022). International surveillance study in acute spinal cord injury confirms viability of multinational clinical trials. BMC Med.

[3] American Spinal Injury Association Expedited ASIA ISNCSCI Exam (E-ISNCSCI), Version 1 (February 2020).

[4] Burns SP, Tansey KE (2020). The Expedited International Standards for Neurological Classification of Spinal Cord Injury (E-ISNCSCI). Spinal Cord.

[5] Zariffa J, Kramer JL, Jones LA (2012). Sacral sparing in SCI: Beyond the S4-S5 and anorectal examination. Spine J.

[6] Noonan VK, Kwon BK, Soril L (2012). The Rick Hansen Spinal Cord Injury Registry (RHSCIR): A national patient-registry. Spinal Cord.

[7] Noonan VK (2023). A Look at Spinal Cord Injury in Canada: Rick Hansen Spinal Cord Injury Registry (RHSCIR) - 2021 SCI data summary. Top Spinal Cord Inj Rehabil.

[8] Walden K, Bélanger LM, Biering-Sørensen F (2016). Development and validation of a computerized algorithm for International Standards for Neurological Classification of Spinal Cord Injury (ISNCSCI). Spinal Cord.

[9] Walden K, Schuld C, Noonan VK, Rupp R (2023). Computer International Standards for Neurological Classification of Spinal Cord Injury (ISNCSCI) algorithms: A review. Spinal Cord.

[10] European Multicentre Study of Human Spinal Cord Injury Clinicaltrials.gov.

[11] Marino RJ, Schmidt-Read M, Chen A (2020). Reliability of S3 pressure sensation and voluntary hip adduction/toe flexion and agreement with deep anal pressure and voluntary anal contraction in classifying persons with traumatic spinal cord injury. J Spinal Cord Med.

[12] Battistuzzo CR, Smith K, Skeers P (2016). Early rapid neurological assessment for acute spinal cord injury trials. J Neurotrauma.

[13] Pelletier-Roy R, Dionne A, Richard-Denis A, Briand MM, Bourassa-Moreau É, Mac-Thiong JM (2023). Validation of a new tool to detect and characterize spinal cord injury in the acute trauma patient: The Montreal acute classification of spinal cord injury. Global Spine J.

[14] Schuld C, Wiese J, Hug A (2012). Computer implementation of the international standards for neurological classification of spinal cord injury for consistent and efficient derivation of its subscores including handling of data from not testable segments. J Neurotrauma.

[15] Kirshblum S, Snider B, Eren F, Guest J (2021). Characterizing natural recovery after traumatic spinal cord injury. J Neurotrauma.

[16] Rupp R, Schuld C, Biering-Sørensen F (2022). A taxonomy for consistent handling of conditions not related to the spinal cord injury (SCI) in the International Standards for Neurological Classification of SCI (ISNCSCI). Spinal Cord.

