# Supplementary material for: Development and Validation of an Algorithm for Item Reduction of the International Standards for Neurological Classification of Spinal Cord Injury Examination to Determine Level and Severity of SCI
Source: Top Spinal Cord Inj Rehabil. 2025 Aug 22;31(3):61–7. doi: 10.46292/sci25-00008 (PMC12376155; doi:10.46292/sci25-00008)
Supplement: Supplementary file 1 [file i1945-5763-31-3-61_s01.pdf]

eAPPENDIX

ISNCSCI Item Reduction Algorithm (IIRA)

Neurological Level of Injury (NLI) -

Motor testing:

- Perform motor testing for the 10 key muscles bilaterally
- Identify the presumed motor level on each side. If motor levels are asymmetric, identify which one is more rostral.
  - "Presumed motor level" is defined as the most caudal muscle that is at least 3/5 with all more rostral muscles testing as normal (5/5), with the following two exceptions: If motor is less than 3/5 at C5, then the presumed motor level is C4; if upper extremity muscles are all normal (5/5) and L2 is less than 3/5, then the presumed motor level is L1. The presumed motor level based on motor testing alone may not be the true motor level, since motor level can be dependent on sensory level.

Sensory testing:

- Begin with light touch testing at C2 on the side with the more rostral presumed motor level. If motor levels are symmetric then begin testing at C2 on either side. Proceed caudally until either:
  - A dermatome score of 1 or 0 is found, **or**
  - The level corresponding to the presumed motor level is reached and has been tested.
- Next, test pinprick on that same side at the most caudal dermatome that had a score of 2 (normal) for light touch in the prior step. Continue testing rostrally for pinprick until two consecutive dermatomes with normal pinprick sensation are identified.
- Next, test light touch on the contralateral side, beginning at the dermatome corresponding to the most caudal dermatome on the first side that tested as normal for both light touch and pinprick for two consecutive dermatomes. Continue testing rostrally until two consecutive dermatomes with normal light touch sensation are identified.
- Finally, test pinprick on this second side, beginning at the dermatome corresponding to the most caudal dermatome that was tested as normal for light touch over two consecutive dermatomes in the prior step. Continue testing rostrally until two consecutive dermatomes with normal pinprick sensation are identified

NLI determination:

- Review the results of the motor and sensory testing in the prior steps. Assume that all untested dermatomes rostral to the tested ones are normal. Use conventional ISNCSCI criteria for NLI determination. The NLI is the most caudal level with normal motor and sensory function bilaterally.

ASIA Impairment Scale (AIS)-

S4-5 Sensory testing:

- Perform sensory testing for light touch and pinprick at S4-5 bilaterally. If any light touch or pinprick sensation is found to be present at S4-5, then the remainder of S4-5 sensory testing can be omitted.

This supplementary digital material accompanies
Top Spinal Cord Inj Rehabil 2025;31(3):61-67
© 2025 American Spinal Injury Association
www.asia-spinalinjury.org
doi: 10.46292/sci25-00008

Digital anorectal exam for deep anal pressure (DAP) and voluntary anal contraction (VAC):

- Perform testing for DAP and VAC only if the AIS classification is dependent on the exam findings, as follows:
  - If some sensation is present at S4-5 and motor is preserved more than 3 levels below the motor level on either side, including non-key muscles, then DAP and VAC can be omitted.
  - If some sensation is present at S4-5 but motor is not preserved more than 3 levels below the motor level on either side, including non-key muscles, then assess for VAC
  - If no sensation is present at S4-5, then assess DAP and VAC.

AIS determination:

- Use conventional ISNCSCI criteria for AIS determination.
- *Note: infrequent requirement for additional sensory testing*
  - This algorithm is designed to identify NLI and AIS. It will omit the sensory testing needed to identify a sensory level that does not define the NLI.
  - This algorithm will not determine a motor level when a more rostral sensory level defines the motor level, i.e., when "motor follows sensory level" for myotomes with no testable key muscles. Rarely, additional sensory testing will be needed to identify a sensory level that determines the motor level, in order to determine whether motor function is spared more than three levels below the motor level.
  - If sensory level classifications are needed, then additional sensory testing can be performed, extending caudally from the previously tested region until the first abnormal dermatome is detected.

Case Classification Examples Using the IIRA

These figures show the testing (red bars) required by the IIRA for NLI and AIS classifications, superimposed on two full ISNCSCI exams from the validation sample EMSCI cases, with NLI and AIS classifications of C7 AIS A and T7 AIS D. The IIRA begins with motor testing of the 10 key muscles bilaterally. Next, light touch sensory testing is performed on one side, beginning at C2 and extending caudally until either an abnormal dermatome is identified or until the level corresponding with more rostral of the 2 presumed motor levels is reached and has been tested. After additional sensory testing and identification of NLI, the minimum amount of sacral (S4-5) and anorectal (DAP and VAC) testing needed for AIS determination is performed.

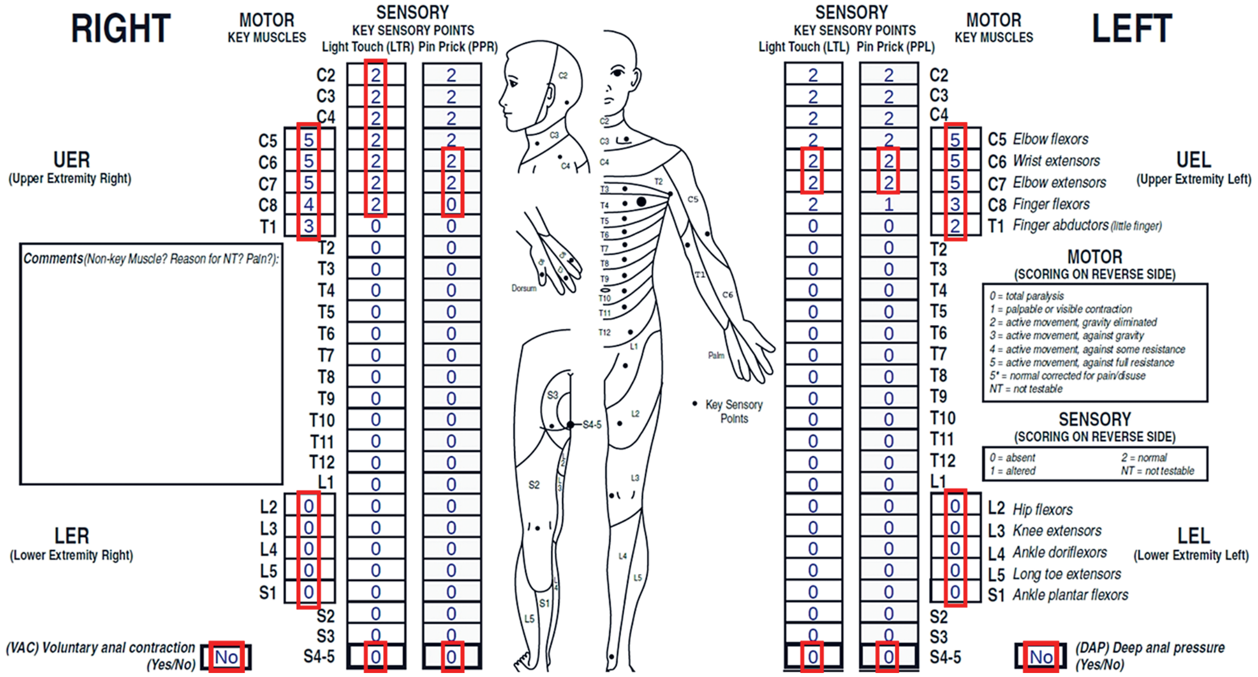

RIGHT | **LEFT**

Level	RIGHT MOTOR KEY MUSCLES	RIGHT SENSORY Light Touch (LTR)	RIGHT SENSORY Pin Prick (PPR)	LEFT SENSORY Light Touch (LTL)	LEFT SENSORY Pin Prick (PPL)	LEFT MOTOR KEY MUSCLES	Key muscle
C2		2	2	2	2		
C3		2	2	2	2		
C4		2	2	2	2		
C5	5	2	2	2	2	5	*Elbow flexors*
C6	5	2	2	2	2	5	*Wrist extensors*
C7	5	2	2	2	2	5	*Elbow extensors*
C8	4	2	0	2	1	3	*Finger flexors*
T1	3	0	0	0	0	2	*Finger abductors (little finger)*
T2		0	0	0	0		
T3		0	0	0	0		
T4		0	0	0	0		
T5		0	0	0	0		
T6		0	0	0	0		
T7		0	0	0	0		
T8		0	0	0	0		
T9		0	0	0	0		
T10		0	0	0	0		
T11		0	0	0	0		
T12		0	0	0	0		
L1		0	0	0	0		
L2	0	0	0	0	0	0	*Hip flexors*
L3	0	0	0	0	0	0	*Knee extensors*
L4	0	0	0	0	0	0	*Ankle dorsiflexors*
L5	0	0	0	0	0	0	*Long toe extensors*
S1	0	0	0	0	0	0	*Ankle plantar flexors*
S2		0	0	0	0		
S3		0	0	0	0		
S4-5		0	0	0	0		

UER (Upper Extremity Right); LER (Lower Extremity Right); UEL (Upper Extremity Left); LEL (Lower Extremity Left)

Comments (Non-key Muscle? Reason for NT? Pain?):

(VAC) Voluntary anal contraction (Yes/No)	No
(DAP) Deep anal pressure (Yes/No)	No

MOTOR (SCORING ON REVERSE SIDE)
0 = total paralysis
1 = palpable or visible contraction
2 = active movement, gravity eliminated
3 = active movement, against gravity
4 = active movement, against some resistance
5 = active movement, against full resistance
5 = normal corrected for pain/disuse*
NT = not testable

SENSORY (SCORING ON REVERSE SIDE)
0 = absent *2 = normal*
1 = altered *NT = not testable*

• Key Sensory Points

eFigure A1a. Based on motor testing, the presumed motor level is C8 bilaterally, which defines the caudal extent of initial light touch sensory testing. Additional sensory testing shows that the NLI is C7. Sensation is absent at S4-5 bilaterally, and DAP and VAC are also absent, confirming AIS A.

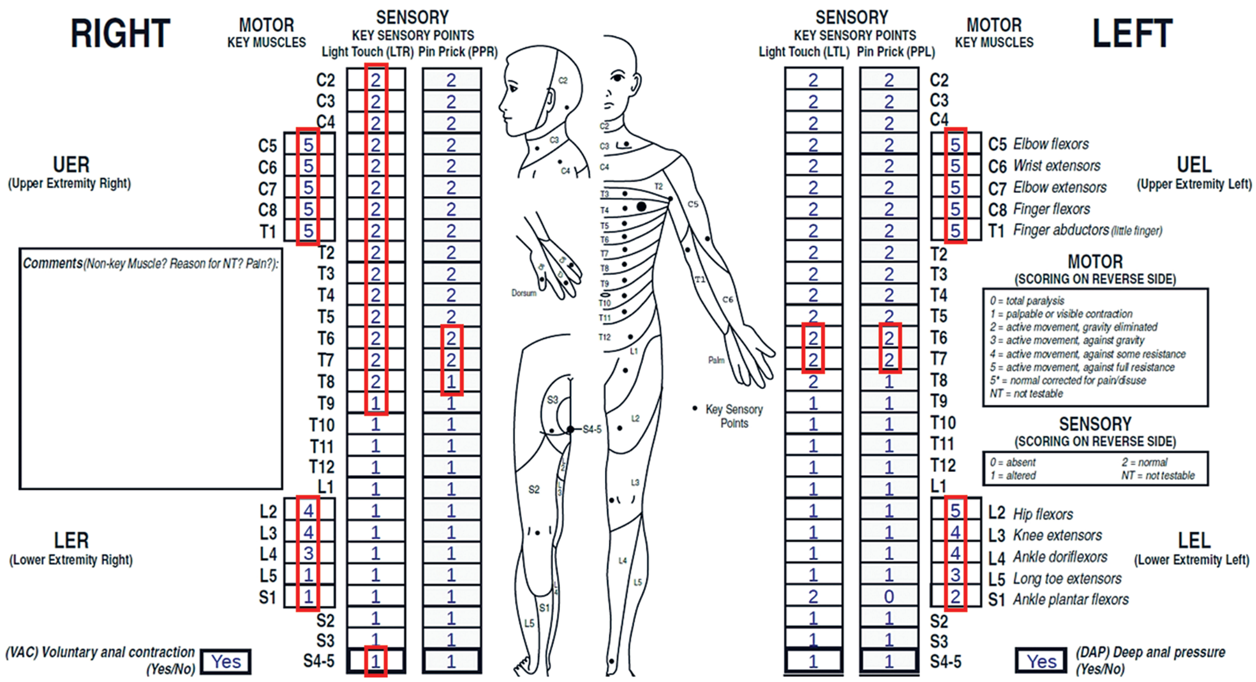

Level	Right Motor	LTR	PPR	LTL	PPL	Left Motor
C2		2	2	2	2	
C3		2	2	2	2	
C4		2	2	2	2	
C5	5	2	2	2	2	5
C6	5	2	2	2	2	5
C7	5	2	2	2	2	5
C8	5	2	2	2	2	5
T1	5	2	2	2	2	5
T2		2	2	2	2	
T3		2	2	2	2	
T4		2	2	2	2	
T5		2	2	2	2	
T6		2	2	2	2	
T7		2	2	2	2	
T8		2	1	2	1	
T9		1	1	1	1	
T10		1	1	1	1	
T11		1	1	1	1	
T12		1	1	1	1	
L1		1	1	1	1	
L2	4	1	1	1	1	5
L3	4	1	1	1	1	4
L4	3	1	1	1	1	4
L5	1	1	1	1	1	3
S1	1	1	1	2	0	2
S2		1	1	1	1	
S3		1	1	1	1	
S4-5		1	1	1	1	

eFigure A1b. Based on motor testing, the most rostral of the 2 presumed motor levels is L2 on the right side. Initial light touch testing on the right is performed caudally only to T9, where abnormal sensation is first detected. Additional sensory testing identifies T7 as the NLI. Presence of light touch at right S4-5 confirms the injury is incomplete. The motor level on the right side is T7, due to the "motor follows sensory level" classification rule, and motor function is preserved in muscles more than three levels below that motor level. Therefore, it is not necessary to check DAP and VAC, since other exam findings confirm the severity is motor-incomplete. Of the 10 key muscles below the T7 NLI, 7 are at least 3/5, so the severity is AIS D.
