# Supplementary material for: Development and Validation of an Algorithm for Item Reduction of the International Standards for Neurological Classification of Spinal Cord Injury Examination to Determine Level and Severity of SCI
Source: Top Spinal Cord Inj Rehabil. 2025 Aug 22;31(3):61–7. doi: 10.46292/sci25-00008 (PMC12376155; doi:10.46292/sci25-00008)
Supplement: Supplementary file 2 [file i1945-5763-31-3-61_s02.pdf]

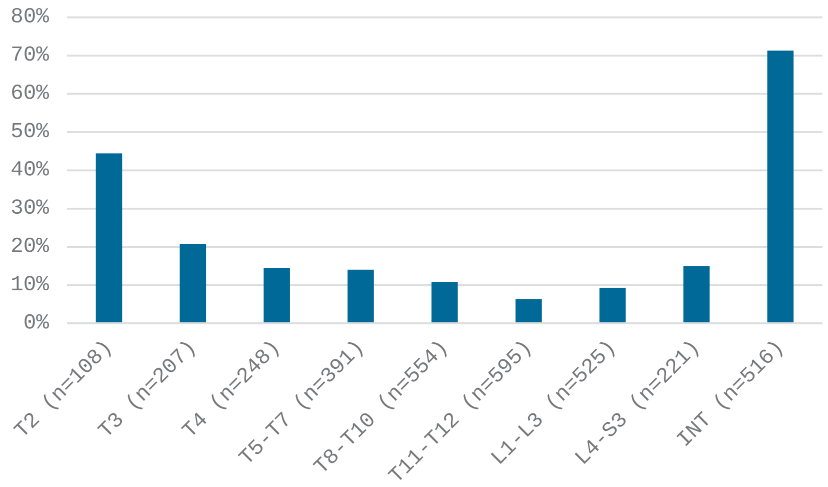

eFigure 1. Frequency of upper extremity motor impairment versus the most rostral sensory level. The percentage indicates the proportion of cases at each most rostral sensory level (or range of levels) that had any motor impairment in the upper extremities. The n listed for each bar is the total number of cases with that level or range of levels. INT = intact (the sensory level designation when sensation is intact from C2 through S4-S5).

This supplementary digital material accompanies
Top Spinal Cord Inj Rehabil 2025;31(3):61-67
© 2025 American Spinal Injury Association
www.asia-spinalinjury.org
doi: 10.46292/sci25-00008
