# Supplementary material for: Development and Validation of an Algorithm for Item Reduction of the International Standards for Neurological Classification of Spinal Cord Injury Examination to Determine Level and Severity of SCI
Source: Top Spinal Cord Inj Rehabil. 2025 Aug 22;31(3):61–7. doi: 10.46292/sci25-00008 (PMC12376155; doi:10.46292/sci25-00008)
Supplement: Supplementary file 3 [file i1945-5763-31-3-61_s03.pdf]

eTable 1. Identification criteria for possible AIS category classification errors

	**AIS with S1 substitution**		
**True AIS**	A	B	C/D
A		vi	iv
B	ii		v
C/D	i	iii	

i = Of the total number of cases, how many cases have an AIS = C or D, with S1 light touch and pinprick on either side = 0/2 AND S1 motor = 0/5 (i.e., if S1 is substituted for anorectal exam then would we be incorrectly classifying as AIS A whereas anorectal exam would tell us patient is actually a C or D)?
ii = Of the total number of cases, how many cases have an AIS = B, with S1 light touch and pinprick on either side = 0/2 (i.e., if S1 is substituted for anorectal exam then would we be incorrectly classifying as AIS A whereas anorectal exam would tell us patient is actually a B)?
iii = Of the total number of cases, how many cases have an AIS = C or D, with no motor function more than three levels below the motor level on each side AND S1 motor 0/5 AND S1 light touch or pinprick on either side = 1-2/2 (i.e., if S1 is substituted for anorectal exam then would we be incorrectly classifying as AIS B whereas anorectal exam would tell us patient is actually a C or D)?
iv = Of the total number of cases, how many cases have an AIS = A, with S1 motor 1-4/5 OR motor function more than 3 levels below the motor level on each side AND S1 light touch or pinprick on either side = 1-2/2 (i.e., if S1 is substituted for anorectal exam then would we be incorrectly classifying as AIS C or D whereas anorectal exam would tell us patient is actually an A)?
v = Of the total number of cases, how many cases have an AIS = B, with S1 motor 1-4/5 (i.e., if S1 is substituted for anorectal exam then would we be incorrectly classifying as AIS C or D whereas anorectal exam would tell us patient is actually a B)?
vi = Of the total number of cases, how many cases have an AIS = A, with S1 motor 0/5 AND S1 light touch or pinprick on either side = 1-2/2 (i.e., if S1 is substituted for anorectal exam then would we be incorrectly classifying as AIS B whereas anorectal exam would tell us patient is actually an A)?

This supplementary digital material accompanies
Top Spinal Cord Inj Rehabil 2025;31(3):61-67
© 2025 American Spinal Injury Association
www.asia-spinalinjury.org
doi: 10.46292/sci25-00008
